# Influence of intermittent flow on removal of organics in a biological activated carbon filter (BAC) used as post-treatment for greywater

**DOI:** 10.1016/j.wroa.2020.100078

**Published:** 2020-11-18

**Authors:** Angelika Hess, Cécile Bettex, Eberhard Morgenroth

**Affiliations:** a Eawag: Swiss Federal Institute of Aquatic Science and Technology, 8600, Dübendorf, Switzerland; b ETH Zürich, Institute of Environmental Engineering, 8093, Zürich, Switzerland

**Keywords:** Biologically activated carbon (BAC), Intermittent flow, Bioregeneration, Biofiltration, Greywater

## Abstract

Highly variable flow has to be expected in decentralized greywater treatment and can lead to intermittent operation of the treatment system. However, few studies have addressed the influence of variable flow on the treatment performance of a biological activated carbon filter (BAC). In this study, we investigated the influence of intermittent flow using small-scale BAC columns, which treat greywater as a second treatment step following a membrane bioreactor (MBR). Three operating strategies to respond to variable flow were evaluated. The activated carbon was characterized before and after the experiments in terms of biological activity and sorption capacity. The performance of the BAC filters was assessed based on total organic carbon (TOC) removal, TOC fractions and growth potential. No significant differences were observed between constant flow compared to on-off operation with intermittent flow over the range of tested influent concentrations. Peaks with high TOC during 24 h periods were attenuated by sorption and biological degradation. Adsorbed TOC was released after switching back to normal concentrations for influent concentrations more than 5 times higher than usually observed, the BAC functioned as a temporary sink. In line with these results, the high influent TOC values led to increased biological activity in the filter but did not influence the sorption capacity. The experiments showed that intermittent flow does not negatively impact the performance of a BAC and that there is no need for additional equalization tanks to buffer the variable flow, for example in household-scale greywater treatment.

## Highlights

• Small-scale BAC tested for post-treatment for greywater with highly variable flow.
• GAC was characterized for both sorption capacity and biological activity.
• On-off operation of flow does not negatively impact BAC performance.
• No bioregeneration was observed during times with stagnant water.

## Graphical abstract

**Figure:**
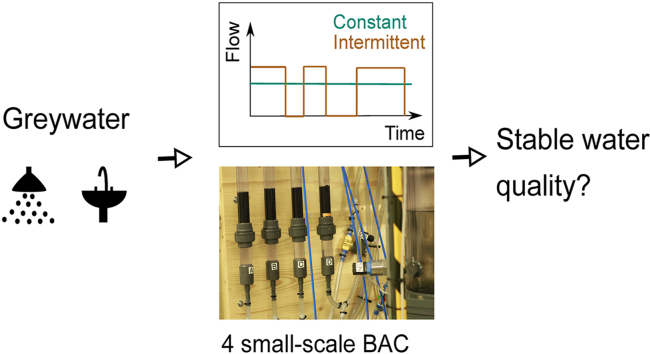

## 1 Introduction

Greywater reuse offers a great water saving potential and in the recent years, interest in greywater treatment and reuse has increased (National Academies of Sciences 2016). In this study, we treat the greywater with a conventional membrane bioreactor (MBR) followed by a biological activated carbon filter (BAC). MBRs are often installed as a first treatment step for greywater treatment (Friedler et al., 2006; Jefferson et al., 2004; Wu 2019). A BAC after the membrane reduces the organics and helps to ensure safe water quality (Ziemba et al., 2019). Additionally, a disinfection step for virus inactivation and removal of pathogens would be necessary to reach water that is hygienically safe. With the chosen treatment configuration we combine physical and biological greywater treatment as suggested in literature (Li et al., 2009).

In drinking water treatment as well as in greywater treatment, hygiene of the treated water needs to be ensured at all times (Benami et al., 2016; Schoen et al., 2017). To ensure high quality not only after the treatment, but also at the consumer, biological stability of the treated water is important (Prest et al., 2016). Biological stability is therefore a requirement to reuse the greywater for higher level purposes that go beyond toilet flushing and irrigation like reuse for showers or washing machines. Assimilable organic carbon (AOC) is considered as a good indicator for growth potential and therefore also for biological stability (Chen et al., 2018). Effluent of an MBR contains mostly slowly biodegradable organic carbon where post-treatment is needed to remove the high molecular weight fractions to approach biological stability (Schurer et al., 2019). BAC filters are effective in reducing the AOC in drinking water (Simpson 2008) and in greywater reuse (Ziemba et al., 2019).

One of the challenges with decentralized greywater treatment is the high variability both in terms of composition (Friedler and Butler 1996; Jefferson et al., 2004) and in terms of flow rate (Fuentes et al., 2018; Penn et al., 2012). The high variability of incoming greywater and the irregular reuse patterns lead to times without flow into the BAC as a secondary treatment step and therefore periods with stagnating water. Additionally, unexpectedly high influent concentrations into the greywater system or worse treatment performance of the MBR as the first treatment step can lead to increased carbon loadings for the BAC. BAC filters can significantly attenuate peaks of biologically degradable compounds and therefore mitigate sudden changes in feed water quality (Ross et al., 2019; Tackaert et al., 2019).

Biological degradation of organic carbon in a BAC can be seen as a multistep process. When the bulk concentration decreases due to biodegradation, DOC from the activated carbon desorbs and is available for further biodegradation (Simpson 2008). Microorganisms survive starvation periods in the BAC thanks to desorption of substrate (Hassan et al., 1998). Due to this desorption, new adsorption sites are available for new DOC entering the BAC. Desorption of biodegradable carbon does increase the sorption capacity for non-biodegradable carbon (Putz et al., 2005). If the microorganisms are adapted to the concentrations in the BAC, the bioregeneration efficiency can be improved (Oh et al., 2011). It is therefore hypothesized that bioregeneration could lead to increased sorption capacity during times of no flow in the BAC filter.

The influence of different operating strategies like empty bed contact time (EBCT), temperature, media type and size on the performance of BAC filters have been tested (Emelko et al., 2006; Kennedy et al., 2015; Moll et al., 1999). McKie et al. (2019) studied the influence of cyclical operation on the biological removal processes in a BAC filter. Cyclical and continuously operated biofilters show similar removal for organics and an increased enzyme activity was measured for cyclical operation. Nemani et al. (2018) showed a modest improvement of DOC removal for cyclical operation and differences in bacterial communities between cyclically operated and constant flow operated filters. While the biological degradation of pollutants during intermittent flow has been studied, there is a lack of understanding how stagnation influences the sorption capacity of a BAC. So far, no studies have been published about different strategies on how to optimally operate the BAC filter during phases with stagnant water.

The objective of this study was to study the effect of intermittent flow and different strategies to deal with it on the effluent water quality of BAC filters. The hypothesis was that intermittent flow leads to higher bioregeneration and therefore higher sorption capacity compared to constant flow. We assessed the differences between BAC filter columns operated with constant and intermittent flow in buffering high influent TOC concentrations. For the different BAC filter columns, we assessed the influence of the operating conditions on the two main removal mechanisms: sorption and biological degradation of organic carbon.

## 2 Material and methods

### 2.1 Greywater treatment system

**Fig. 1:**
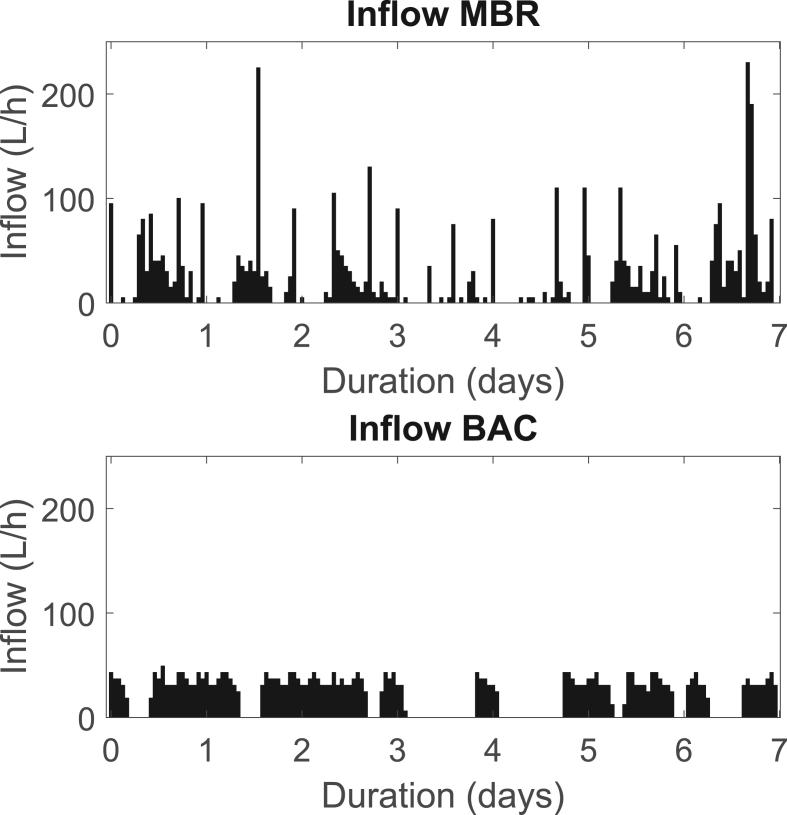
Measured inflow into MBR and BAC over the duration of a typical week.

The greywater treatment system is located in the Water Hub at NEST. NEST is a modular research and office building in which the different wastewater streams (grey-, yellow- and blackwater) are collected separately and treated in the Water Hub (Etter et al., 2016). The light greywater from showers and washbasins collected in the building is treated by a two-step process: a membrane bioreactor (MBR) followed by a biological activated carbon (BAC) filter. The flow into this household-scale greywater treatment is highly variable (Fig. 1). The MBR with a median hydraulic retention time of 13 and 17 h in the first and second tank, respectively, cannot buffer all of the variable flow. The on/off operation of the filtration in the MBR leads to intermittent flow into the BAC. In this study, we examined the effect of intermittent flow on the BAC as a secondary treatment step.

### 2.2 Experimental setup

Small-scale column studies were performed with four side-by-side BAC filter columns (3 cm diameter). The feed water was collected after a full-scale MBR, treating on average 413 L/d of light greywater coming from showers and wash basins (average composition of the MBR permeate over a 2 years period: TOC: 5.2 mg/L, NO_3_–N: 9.7 mg/L, NH_4_–N: 0.3 mg/L, PO_4_–P: 2.0 mg/L). A 50 L polyethylene-tank was automatically emptied and refilled every one to two days with fresh MBR permeate. The water was pumped from the feed tank to the four small-scale columns. The times of stagnation and flow were chosen in a way to represent the same statistical characteristics as typically observed for the inflow of the full-scale BAC (Fig. 1). The stagnation times varied between 9 and 30 h and times with flow between 7 and 31 h. The same sequence of flow and no flow was repeated every week. The applied pattern is shown in Supporting information S1.

The experiments were conducted in two rounds, first comparing different operating strategies starting with fresh GAC (Experiment 1) and second, focusing on the best strategy from the first round using already exhausted GAC (Experiment 2). The exhausted GAC was taken from the top 10 cm of the full-scale BAC. At this time, more than 30′000 empty bed volumes $\left(EBV=\frac{V_{\text{treated water}}}{V_{Filter}}\right)$ have been treated in the top part of the full-scale BAC (4′000 EBV if examined over the whole filter bed). Long-term measurements in the full-scale filter show that the top part has reached “period C″ as defined in Simpson (2008) (data not shown). The sorption capacity for this GAC is significantly reduced compared to virgin GAC (Supporting information S6). In Experiment 1, the empty bed volumes per week were kept constant while for Experiment 2, the empty bed contact time (EBCT) was kept constant for all columns. Table 1 summarizes the hydraulic conditions in the columns for the two experiments. The hydraulic conditions are representing the conditions we have in the top part of the full-scale BAC operated for greywater treatment in the Water Hub.

**Table 1:** Operating conditions for the different columns.

Experiment	Condition	Flow pattern	Filtration rate (m/h)	EBCT (min)	EBV/week	GAC
**Experiment 1**	Submerged intermittent	Intermittent^a^	0.8	7	644	Fresh
	Recirculation intermittent	Intermittent^a^	0.8	7	644	Fresh
	Dry intermittent	Intermittent^a^	0.8	7	644	Fresh
	Constant	Constant	0.4	16	644	Fresh
**Experiment 2**	Intermittent A & B^b^	Intermittent^a^	0.3	12	428	Exhausted
	Constant C & D^b^	Constant	0.3	12	857	Exhausted

a Intermittent flow: periods with flow and no flow alternate. Typical patterns as observed for full-scale BAC.

b BAC B & D were sacrificed to analyze biological activity and sorption capacity after the period of normal influent while BAC A & C continued to operate during high influent TOC conditions.

#### 2.2.1 Experiment 1: small-scale columns with 4 different operating strategies

The filtration media for all four columns was fresh GAC (Chemviron F-400). All columns had a filter height of 10 cm, the filtration rate was chosen such that the weekly loading for all columns was equal. Three different operating strategies during times with no flow were tested: (i) a minimum water head was maintained on top of the filter bed (submerged intermittent), (ii) the effluent was recirculated through the column during no influent to maintain constant hydraulic conditions (recirculation intermittent), and (iii) the filter bed was drained when there was no influent (dry intermittent). A column with continuous inflow was operated for comparison. The columns with intermittent flow had flow during 50% of the time. Water samples were collected twice a week in the feed tank and in the effluent of all four columns. The columns were operated over 56 days, which corresponds to around 5000 EBVs.

#### 2.2.2 Experiment 2: small-scale columns with 2 different operating strategies

##### 2.2.2.1 Normal influent concentrations

The filtration media for all four columns was GAC (Chemviron F-400) obtained from the top 10 cm of the full-scale BAC filter from the Water Hub at NEST that was running for more than one year. All four columns were operated with the same filtration rate and a filter bed height of 5 cm. The columns with intermittent flow had flow during 50% of the time. Nonetheless, all four columns should have received the same total TOC loading at the time when the high influent experiment started. Therefore, the columns with constant flow were taken into operation when the columns with intermittent flow had already treated 860 EBV. The GAC for all columns was sampled at the same day and then stored in the dark at 4 °C for the columns being started later. Every filling of the feed tank with MBR permeate was sampled for TOC. With this measurements, the TOC load the columns received was calculated to make sure all four columns receive the same total TOC load over time. Additionally, water samples were collected in the effluent of all four columns. At the time, when all four columns had received the same TOC loading, the experiment with high influent concentration was started and columns B and D were analyzed for sorption capacity and biological activity.

##### 2.2.2.2 High influent concentrations

The effect of high influent loading was tested on two columns, both with the same previous loading, one operated with intermittent flow, the other with constant flow. To increase the influent TOC concentration, 10 L of raw greywater were sampled, filtered through a 0.45 μm filter (Satorius, cellulose nitrate filter) and stored at 4 °C until use. With the filtered raw greywater, a failure of biological TOC degradation in the MBR was mimicked. The influent for the columns was kept at high concentration for 24 h and was afterwards switched back to MBR permeate. The influent concentration was increased to 20, 40, and 70 mg/L TOC.

### 2.3 GAC characterization

#### 2.3.1 Quantification of biomass on GAC

The biomass was quantified similar as described in Velten et al. (2007). In short, 1 g (wet weight) of GAC particles were transferred in a muffled glass vial with 10 mL of 0.2 μm filtered (Millex-GP, Merck Milipore Ltd) tap water. The sample was sonicated for 3 min (Bandelin Sonorex, 320 W and 35 kHz), 9 mL supernatant was collected in a separate muffled glass vial and the sample was refilled with 9 mL of 0.2 μm filtered (Millex-GP, Merck Milipore Ltd) tap water. This step was repeated in total three times to recover more than 98% of the ATP. The collected supernatant was vortexed and 0.5 mL of the sample were incubated at 38 °C for at least 4 min. 100 μL of sample together with 100 μL of BacTiter-Glo™ Microbial Cell Viability Assay (Promega Corporation, Madison, WI, USA), were measured for relative light units (RLU) and converted to ATP concentrations using a calibration curve (Supporting information S4).

#### 2.3.2 Sorption capacity

Methylene blue (Fluka, 66720, 99%) was used as a competing adsorbent to characterize the sorption capacity. Previous experiments with GAC from the full-scale BAC filter showed that the adsorption of methylene blue is sensitive to the remaining sorption capacity on the GAC (Supporting information S6). 1 g of GAC (wet weight) was added to 500 mL methylene blue solution (concentration: 400 mg/L) in a clean 1 L Schott bottle. The bottles with the samples were shaken with an overhead shaker for 3 d at room temperature. Samples at the beginning and after 3 d were analyzed for methylene blue. The decrease in methylene blue over time per added mass of GAC was used to characterize the sorption capacity. Sorption capacity was determined before experiment 2 started and then before and after high influent concentrations.

### 2.4 Growth potential

Growth potential measurements were based on the work of Hammes and Egli (2005) and Prest et al. (2016). In short: 20 mL of 0.2 μm filtered (Millex-GP, Merck Milipore Ltd) water sample was inoculated with 20 μL unfiltered sample as inoculum and incubated and agitated at room temperature (22 ± 1 °C) for 5 d. All glassware was muffled at 450 °C for 4 h prior to use. Afterwards, the samples were analyzed for total cell counts (TCC) using SYBR® Green I stain (ThermoFisher Scientific, Waltham, Massachusetts, USA) and a flow cytometer (Cytoflex, Beckman Coulter, Brea, California, USA) with 60 s reads on 60 μL samples. The measured cell counts were not converted by a constant factor to AOC values but reported directly as growth potential.

### 2.5 Chemical analyses

TOC was measured using a total organic carbon analyzer (Shimadzu TOC-L CSH, Kyoto, Japan). Nitrate and Phosphate were measured by ion chromatography (Metrohm 930 Compact IC Flex with column Metrosep A SUPP 7, Herisau, Switzerland). Ammonium was measured with Hach Lange kits (LCK 304, Hach, Loveland, Colorado, USA). Methylene blue was measured with a spectrophotometer (Hach, DR 3800) at 668 nm.

#### 2.5.1 LC-OCD

After in-line filtration (0.45 μm polyethersulfone filter) and acidification, DOC was characterized as different weight fractions by size exclusion chromatography (SEC) and quantified by both ultraviolet (UV) and organic carbon (OC) detectors (SECOCD, DOC Labor Dr. Huber, Karlsruhe, Germany). Details about the SEC-OCD instrument and the analytical method can be found in Huber et al. (2011). The UV detector (Knauer K- 200) provided an online UV signal and OC was detected by an infrared (IR) detector after oxidation of DOC to CO_2_ in a Graentzel Thin-Film Reactor. Chromatograms were interpreted with customized software (Fiffikus, Huber et al. (2011)). A phosphate buffer was used as the eluent (24 mM, pH 6.6) and the flow rate was set at 1 mL/min. All glassware was muffled at 450 °C for 4 h.

### 2.6 Data analysis

The different operation strategies were analyzed for differences with ANOVA (IBM SPSS Statistics 24). The data set from experiment 2 was divided into two data sets: data during normal influent conditions and data during elevated influent concentrations.

## 3 Results and discussion

### 3.1 Different operating strategies (Experiment 1)

**Fig. 2:**
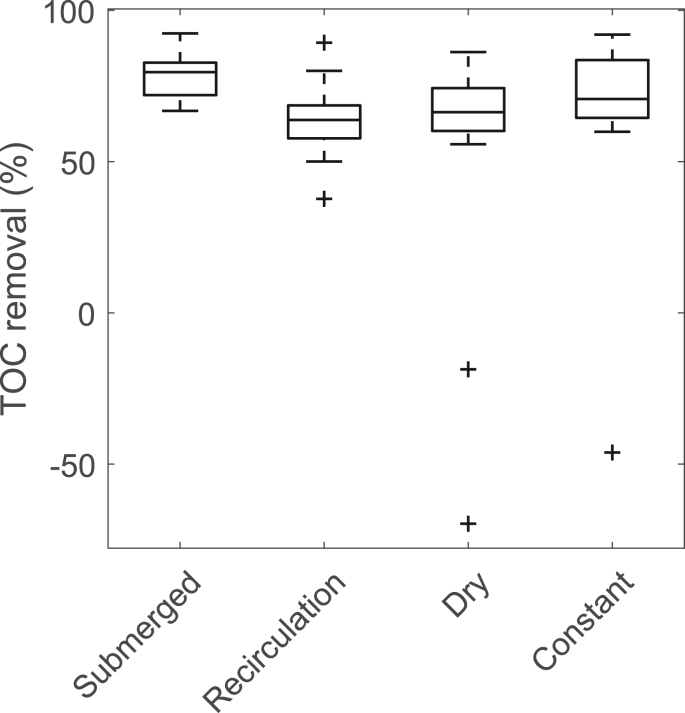
TOC removal for the four differently operated columns (n = 21). The operating strategies were: (i) the filter bed was always submerged with stagnating water, (ii) during times with no influent, the effluent of the filter was recirculated through the filter to maintain constant hydraulic conditions, and (iii) the water was drained providing for aeration. The whisker of the box plot extend the box by maximum 1.5 times the interquartile range.

In Experiment 1, four lab-scale BAC filters were operated side-by-side with different operating conditions and TOC was monitored (Fig. 2). Three strategies in response to times with no flow due to the intermittent regime were evaluated and compared to a BAC with constant influent flow. High TOC removal was observed independently of the operating strategy. The average influent concentration during the experimental period of 3.7 mg/L TOC was reduced to average TOC effluent values of 0.7 mg/L for the column with constant water head, to 1.1 mg/L TOC for the column with recirculated flow and the one running dry during no flow and to 0.9 mg/L TOC for the column with constant inflow. Comparing the three different intermittent flow strategies, one can see that the BAC filter that was always submerged showed the best TOC removal performance; but differences between the filters were not significant (p = 0.06). The columns were run for around 5000 EBV. A comparison of the removal after 5000 EBVs with literature shows good removal even tough in this experiment the influent TOC was higher than in literature (Gibert et al., 2013; Han et al., 2013; Velten et al., 2011b). The filter that was dry during no flow showed negative removal right after the flow started again (two samples), this could for example be caused due to washout of particles caused by the rapid start up (Colton et al., 1996). Neither for the fresh GAC at the beginning of the experiment nor for the more exhausted GAC towards the end of the experiment, the differences in TOC removal between the columns were significant. TOC values over time are shown in supporting information S2. The experiment showed that more complicated operating strategies like recirculation of the effluent did not improve the removal performance. Based on these results, constant flow was compared to submerged intermittent flow in Experiment 2 in more detail.

For all four BAC filters, the ATP at the end of the experiment was highest on top of the filter, ranging from 3.5·10^−6^ gATP/gGAC to 8.8·10^−6^ gATP/gGAC, both as was expected based on literature (McKie et al., 2019; Velten et al., 2011a) (ATP results shown in supporting information S5). The lowest ATP concentration in the top section was measured for the filter that was dry during times of no inflow. For the other three filters, the ATP values on the top of the filter bed were close to each other. From 2 to 10 cm depth of the filter bed, all four columns showed very similar ATP values. From these values it can be concluded, that biomass established on all four columns.

### 3.2 TOC removal during normal influent (Experiment 2)

**Fig. 3:**
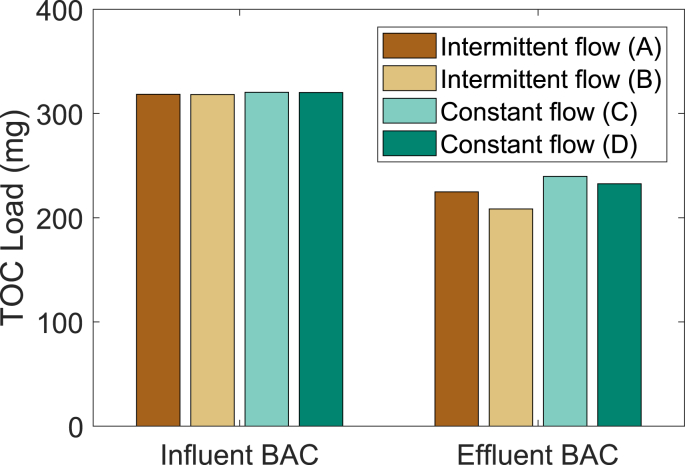
TOC load for the different columns over a period of 36 days for intermittent flow respectively 21 days for constant flow.

Even though Experiment 1 showed no significant differences between the different operating strategies, the submerged regime performed slightly better. Therefore, in Experiment 2, two submerged columns with intermittent flow were compared to two columns receiving constant influent flow in more detail. All columns received MBR permeate as influent. Columns C and D (operated with constant flow) were started 15 days later than columns A and B (operated with intermittent flow). Operation was synchronized to achieve the same TOC loading for all columns by the end of the experimental period (Fig. 3). The observed overall effluent loading was very similar for all four columns regardless of the difference in operation. The effluent load over the time with normal influent concentration was 225 and 208 mg TOC for the columns with intermittent flow. For the columns with constant flow, the effluent load was 240 and 233 mg TOC. There are no significant differences between the effluent water quality in terms of TOC for the four different columns (p = 0.847, Supporting information S2 for TOC, growth potential and TOC fractions over time). These results are in agreement with the results of Nemani et al. (2018) and McKie et al. (2019) who showed that the EBCT is more relevant for the pollutant removal than the operation strategy. In Experiment 2, EBCT was the same for intermittent (columns A and B) and constant (columns C and D) flow during times of filtration (Table 1).

**Fig. 4:**
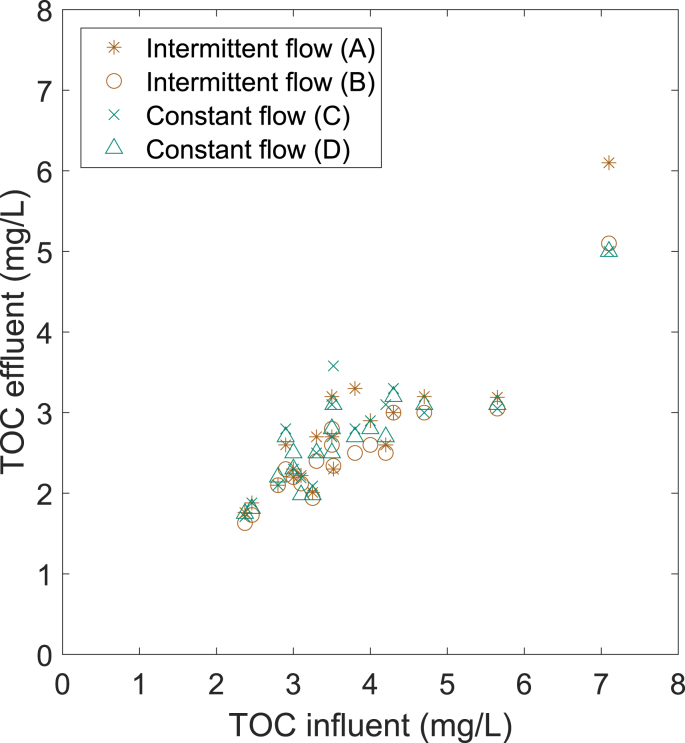
Effluent TOC concentrations for the different columns shown against the influent TOC. The data is shown for the period with all four columns running and receiving normal MBR permeate as influent.

In addition to looking at the overall removal, it is important to assess how the different BAC filters react to varying influent TOC. For decentralized greywater treatment systems, the TOC concentrations in the raw greywater vary due to user behavior in the building (Friedler 2004). The BAC is used as post-treatment after the MBR and therefore, the TOC variations in the influent of the BAC are caused both by variable concentrations in the raw greywater and variable removal performance in the preceding MBR. During the experimental period, the influent TOC concentrations were on average 3.7 mg/L, varying between 2.4 mg/L and 7.1 mg/L. The TOC removed in the BAC during the same period was on average 1.0 mg/L, varying between 0.2 mg/L and 2.6 mg/L. All four columns removed more TOC for higher influent TOC concentrations (Fig. 4). None of the columns performed superior compared to the others in buffering high TOC influents.

**Fig. 5:**
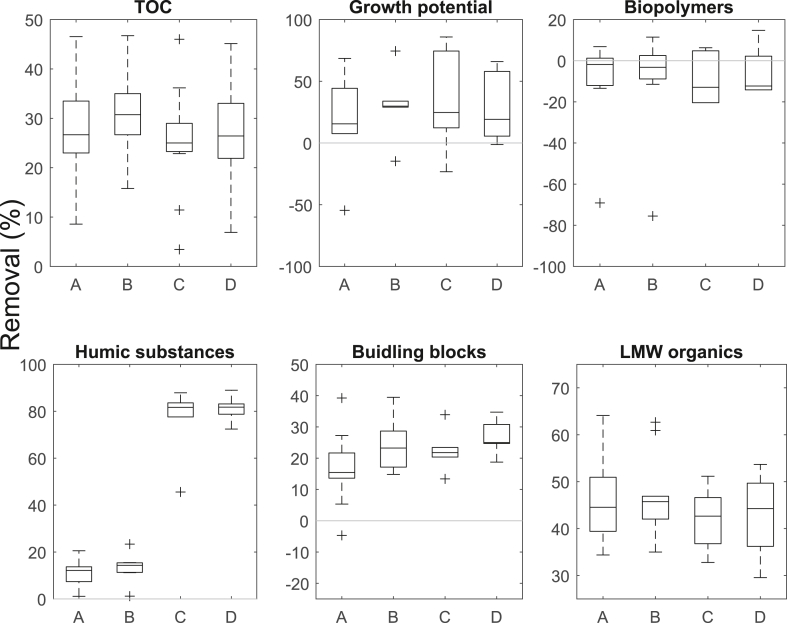
Growth potential and the different fractions of organic carbon during the normal influent. A and B: Intermittent flow (n = 31 for TOC, n = 10 for SEC fractions, and n = 6 for growth potential), C and D: constant flow (n = 19 for TOC, n = 6 for SEC fractions and growth potential). The whisker of the box plot extend the box by maximum 1.5 times the interquartile range. For visual support, a grey line is drawn at 0% removal.

The TOC removal was further assessed by analyzing the removal performance in terms of growth potential and the different weight fractions of the organic carbon determined with SEC (Figure 5). All four columns removed similar fraction of the overall TOC, on average 27%, 31%, 24% and 27% for columns A, B, C, and D, respectively. From Fig. 5, it can be seen that the removal performance varied between less than 10% to nearly 50%, with around 80% of the values between 20% and 40% removal. The removal of growth potential was similar for all four columns varying between −55% and 86% removal (Fig. 5). On average, between 16% and 33% of the growth potential was removed in the BAC filters. For biopolymers (no significant removal), building blocks (20–30% removal) and low molecular weight (LMW) organics (around 50% removal) both intermittent and constant flow show very similar results. These results are in the range previously reported for BAC filters (Gibert et al., 2013, Velten et al. (2011b)). Compared to Experiment 1 with fresh GAC, the TOC removal was lower in Experiment 2 with the exhausted GAC from the full-scale BAC. These results indicate that sorption still played a role at the end of Experiment 1, leading to the better removal performance. Literature reports very little AOC removal for filters with similar amount of treated bed volumes (Velten et al., 2011a). In this work we do not report AOC, but the removal of growth potential that can be used as a comparable indicator as AOC since they are linked by a conversion factor. There is a significant difference between the columns with intermittent and constant flow for the removal of the humic substances. The low removal for the intermittent flow corresponds with values from previous research done (Chen et al., 2016; Gibert et al., 2013; Velten et al., 2011b). The removal of humic substances (on average nearly 80%) for the columns with constant flow is unexpectedly high. Humic substances are known to adsorb on GAC (Velten et al., 2011b). Humic substances are not easily biodegradable but are still biodegraded, mainly if there is no easily biodegradable substrate available (Volk et al., 1997). It is not clear if higher biodegradation or adsorption caused the significant difference in the removal of the humic substances in this experiment. Because the humic substances make on average only 25% of the influent TOC, the difference in removal of humic substances does not show in the TOC values. Overall, the removal performance of the different fractions is little influenced by the change of operating conditions.

### 3.3 TOC removal during high influent (Experiment 2)

Challenging the BAC columns with high influent TOC concentrations is interesting from two points of view. Firstly, it is of practical value to observe if and how fast the filters can recover. Secondly, the response to the high influent can give an indication about the relevance of sorption and biological degradation. High influent TOC concentrations of 20, 40, and 70 mg/L (compared to an average normal influent TOC of 3.7 mg/L) were fed to the BAC columns for periods of 24 h each time. During high influent concentrations, both columns (A and C) received constant flow, while between high influent periods, there were periods with stagnating water in column A.

**Fig. 6:**
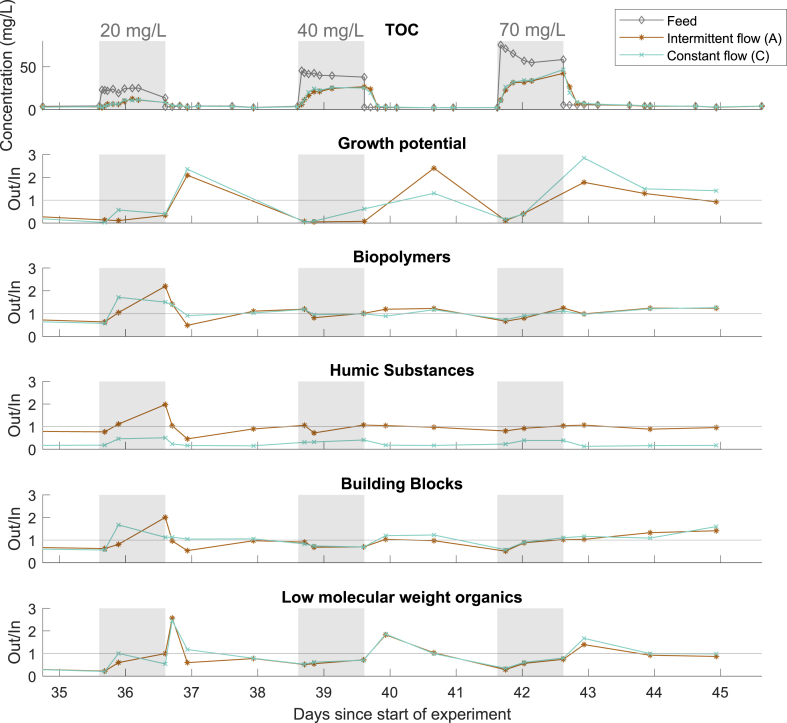
Growth potential and the different fractions of organic carbon during the high influent. The periods with high influent TOC are highlighted in grey.

The removal of TOC, growth potential, and weight fractions of the TOC were measured over time during the experiment with elevated influent TOC concentrations. This data allows to have a closer look at the processes occurring in the BAC filter (Fig. 6). The results for intermittent flow and constant flow are very similar, both for the growth potential, as well as for most of the different TOC fractions. The only exception with significant differences are the humic substances. Compared to periods with normal influent, the removal of the high molecular weight organics such as biopolymers and humic substances is lower. However, for these compounds, the dynamics of the influent concentrations do not show in the effluent. In contrast, the growth potential and the low molecular weight organics show a delayed response with leaking to the influent dynamics. The assimilable carbon measured with the growth potential and LMW organic carbon leak out of the column after the influent is set again to normal influent concentrations. During this period, higher concentrations of LMW organics are measured in the effluent than in the influent. LMW organic carbon can represent the metabolic products of microorganisms living on the GAC surfaces (Velten et al., 2011b). One possible explanation is that increased biological activity during high influent leads to higher metabolic activity in the BAC. This rapid increase in metabolic activity leads to the increased concentrations in low molecular weight organics. The influent TOC consist on average of 45% low molecular weight organics (SD = 16%), so this delayed response to the high influent should not be neglected. Typical chromatograms are shown in supporting information S3. LMW organics do not adsorb so well and are mostly removed by biodegradation (Matilainen et al., 2006; Velten et al., 2011b). This highlights the importance of biological degradation for the overall TOC removal, because the influent TOC consists to 45% of LMW organics.

**Fig. 7:**
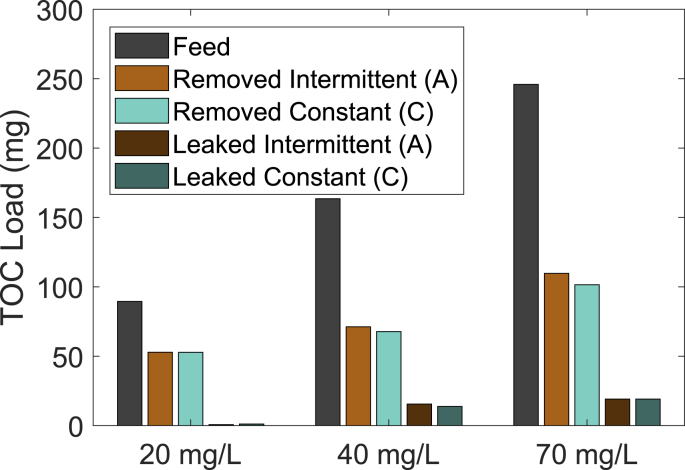
TOC load in the influent of the columns, the removed TOC during the 24 h with high influent and the TOC leaking the 24 h after switching back to normal influent. Both the removed and the leaked TOC are calculated as the difference between the influent and effluent TOC.

As for the normal influent conditions, the TOC removal in terms of overall loading is very similar for intermittent and constant flow (Fig. 7). During influent TOC concentrations of 20 mg/L, a total load of 89 mg was fed to the columns; both columns previously operated with intermittent or constant flow removed 52 mg of TOC (58% of the influent load). Leaking is calculated for the 24 h after switching back to normal influent as the integrated difference between the effluent and the influent TOC over 24 h. Only 0.7, respectively 1.1 mg of TOC leaked out of the BAC. This indicates that the BAC filters can handle influent concentrations up to 20 mg/L very well. For influent concentrations of 40 and 70 mg/L TOC, the effluent concentrations were higher than the influent for a while after switching back to normal influent concentrations (Fig. 6). For influent concentrations of 40 mg/L, the effluent concentrations of both columns are below the influent concentrations again after 24 h, which equals to more than 100 EBVs. For 70 mg/L influent TOC, the effluent concentrations are below the influent after 24 h for the intermittent flow and after 48 h for constant flow. The higher the influent load, the higher the removed load. For 40 and 70 mg/L influent TOC, 34% and 37% of the influent loading are long-term removed in the BAC (calculated as removed – leaked TOC) (Fig. 6). This removal indicates that TOC is biologically degraded (Sakoda et al., 1996). Bourneuf et al. (2016) evaluated the response of peak loading and observed only attenuation of peaks but no long-term removal indicating the GAC acting as a temporary sink. In this study, some desorption is observed, but a considerable amount of TOC is removed for long-term. Therefore, this data indicates some sorption-desorption for the high influent, but biological degradation as the main removal mechanism.

### 3.4 Characterization of GAC (Experiment 2)

#### 3.4.1 Biomass on GAC

**Fig. 8:**
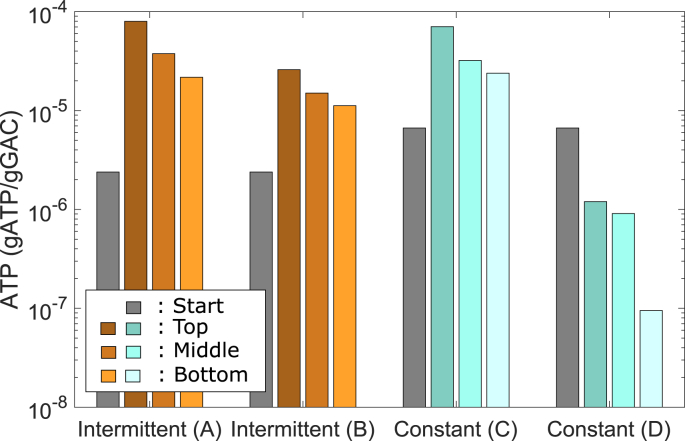
ATP on the GAC for the different columns before start of the experiment (grey) and at different section in the column after the experiment (color gradients: top, middle and bottom of the filters). The ATP for columns B and D was measured after normal influent concentrations (day 35), for A and C after the high influent TOC (day 45). (For interpretation of the references to color in this figure legend, the reader is referred to the Web version of this article.)

ATP concentrations were measured before the operation of the columns started and after the experiments at three different heights over the filter bed to characterize the biological activity (Fig. 8).

Before starting the operation of the columns, the ATP on the GAC was uniform over the filter bed. Already after 35 days and 1350, and 1150 EBV for columns B and D, respectively, a clear gradient over the filter bed is visible for normal influent TOC concentrations for both intermittent and constant flow. The highest ATP concentrations are measured at the top of the filter bed and the lowest at the bottom. After the normal influent, the ATP on the column with intermittent flow (B) is higher than for the column with constant flow (D). The ATP content increased significantly after the high influent for both intermittent and continuous flow (columns A and C). Generally, the measured ATP concentrations are high compared to values reported in literature. For drinking water biofilters, 10^2^–10^3^ ng ATP/cm^3^ media (corresponds to around 10^−7^ to 10^−6^ g ATP/g GAC) are reported as typical ATP concentrations, but the reported values going up to nearly 2 ·10^4^ ng ATP/cm^3^ (Pharand et al., 2014). ATP concentrations increase with influent TOC concentrations. The values reported by Pharand et al. (2014) were for TOC concentrations up to 6 mg/L so that relatively high ATP concentration were to be expected for BAC columns receiving MBR effluent. The increased ATP concentration after the high influent TOC indicates additional biological activity due to the high TOC concentrations (Velten et al., 2007) and therefore increased biological degradation of the additional TOC. The ATP and TOC removal data shows that the biological activity and the biological degradation adapts very rapidly to the increased influent TOC and that a relevant portion of the extra load was thereby degraded.

#### 3.4.2 Sorption capacity

**Fig. 9:**
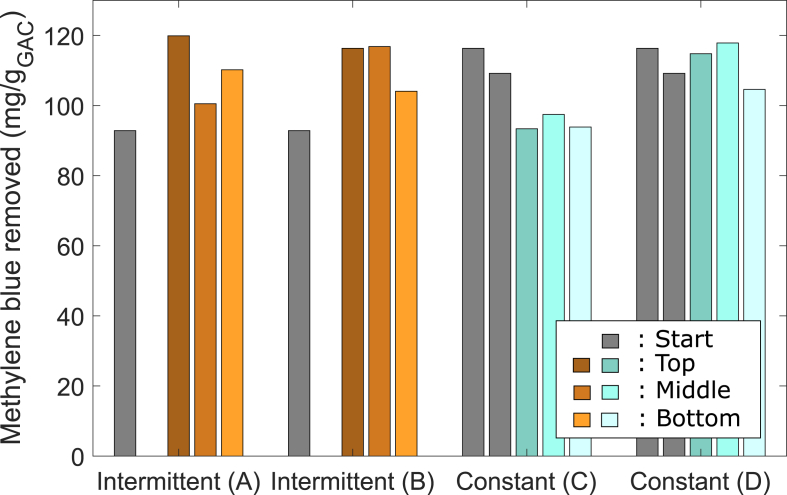
Methylene blue removed as a measure for the sorption capacity for the different columns. The sorption capacity for columns B and D was measured after normal influent concentrations, for A and C after the high influent TOC. The sorption capacity for virgin GAC is 217 mg/gGAC. For comparison, the removed methylene blue was also measured before the columns were started (exhausted GAC from full-scale BAC).

Sorption is the second important removal mechanism in a BAC filter besides biodegradation. The sorption capacity was characterized with methylene blue before operation of the columns and after the experiments (Fig. 9).

Different from the biological activity, no gradient over the filter column is observed for the sorption capacity. The sorption capacity is lower compared to virgin GAC (217 mg/gGAC) for all four columns. No changes of the sorption capacity are observed for different sampling times (Column A vs. B and C vs. D). In comparison, in the full-scale BAC a change of sorption capacity over the filter bed was observed (Data from full-scale BAC in Supporting information). The comparison of the column with intermittent and constant flow after normal influent TOC concentrations (columns B and D) shows no relevant differences in sorption capacity. No difference in sorption capacity indicates no significant influence of bioregeneration during the operation of these columns. The high influent concentrations (Columns A and C compared with B and D) did not significantly influence the sorption capacity, indicating that also for high influent, biological degradation is the main removal mechanism. These results are in line with the results presented in previous sections showing that not as much TOC leaks as entered in the BAC. Intermittent flow and constant flow BAC show the same sorption capacity after the experiments and same attenuation of peaks during high influent TOC. These results indicate that no significant bioregeneration of the GAC happens during times of no flow.

### 3.5 Practical relevance

For the application in decentralized greywater treatment, treatment processes need to be sufficiently robust to provide good quality effluent even with highly variable influent concentrations and load. Furthermore, these systems should be as simple as possible in operation and maintenance. A system that can deal with this variability without intervention of humans is therefore needed. Our comparison of different operating strategies showed that a filter that is constantly submerged in water is therefore the most suitable solution, both in terms of operation and effluent water quality. In the BAC columns, the biological activity adapted rapidly to high influent TOC with an increase in the biological degradation rate. If the influent is extremely high (more than 10 times higher than normal), some time is needed until the system recovered and previous treatment performance is reached. This recovery time is longer for biological stability than for overall TOC removal. Therefore, operators of such systems should be careful when they assess the effluent water quality solely on TOC. Such high influent concentrations can be also of relevance for drinking water treatment, for example when a treatment step breaks (Ross et al., 2019).

Some guidelines for decentralized greywater treatment suggest equalization tanks to buffer the load and increase the stability of the treatment system (Sharvelle et al., 2017). We showed that BAC filter can effectively buffer influent loadings and that the effluent water quality is not worse because of intermittent flow. Our study therefore showed that for the chosen treatment configuration, no extra equalization tanks are needed.

The conducted experiments with small-scale columns allow to draw conclusions for full-scale systems that have an established biological activity and no long-term sorption capacity left. The results should not be applied to activated carbon filters where sorption is the main removal mechanism.

## 4 Conclusions

The main conclusions from this work are:

- Building scale greywater treatment is characterized by variable loading and flow. Stable operation and good effluent quality in terms of organic carbon, organic carbon fractions and growth potential is possible with such intermittent flow. Alternative operating strategies to counteract effects of intermittent flow in the BAC did not result in significant improvement of performance. Therefore, the simplest approach (submerged intermittent) with stagnant water on top of the filter bed is suggested. There is no benefit of using additional buffer tanks for hydraulic buffering of the influent to the BAC.
- Peak TOC influent concentrations (5–20 times the normal influent TOC) were attenuated in the BAC due to sorption and biological degradation. Overall, TOC was mostly biodegraded and even during high influent concentrations, sorption played a minor role.
- No differences in TOC peak attenuation and sorption capacity were observed for different operating strategies. These results indicate that bioregeneration of sorption capacity did not play a relevant role during intermittent flow.

## Declaration of competing interest

The authors declare that they have no known competing financial interests or personal relationships that could have appeared to influence the work reported in this paper.
