# Supplementary material for: Influence of intermittent flow on removal of organics in a biological activated carbon filter (BAC) used as post-treatment for greywater
Source: Water Res X. 2020 Nov 18;9:100078. doi: 10.1016/j.wroa.2020.100078 (PMC7704463; doi:10.1016/j.wroa.2020.100078)
Supplement: Multimedia component 1 [file mmc1.pdf]

Influence of intermittent flow on removal of organics in a biological activated carbon filter (BAC) used as post-treatment for greywater

Supporting information

Angelika Hess [a,b], Cécile Bettex [a], Eberhard Morgenroth [a,b]*

[a] Eawag: Swiss Federal Institute of Aquatic Science and Technology, 8600 Dübendorf, Switzerland

[b] ETH Zürich, Institute of Environmental Engineering, 8093 Zürich, Switzerland

**Corresponding Author. Email address: Eberhard.Morgenroth@eawag.ch*

Submitted to Water Research X on July 15, 2020

S 1 On/Off Operation

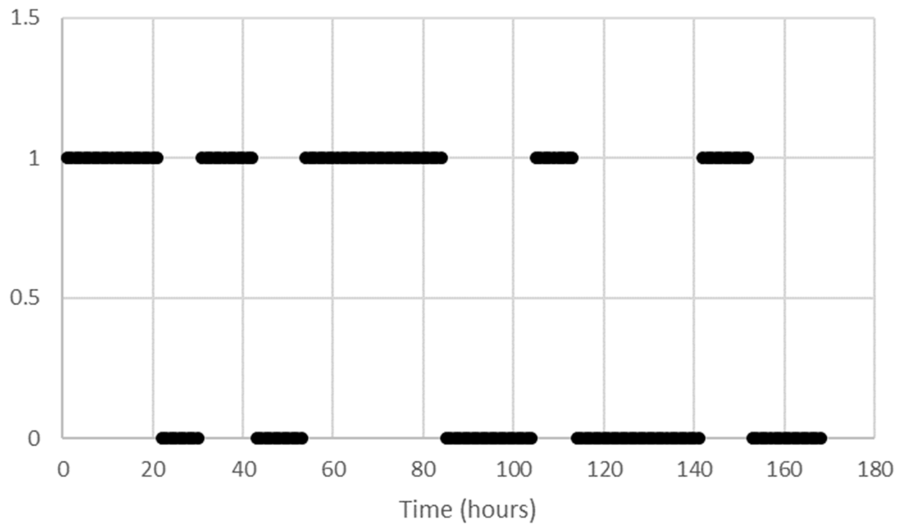

Figure S1: Patterns for On-Off Operation of small-scale columns. 1 indicates influent while 0 indicates no influent flow for columns receiving intermittent flow.

S 2 TOC measurements

2.1 Experiment 1

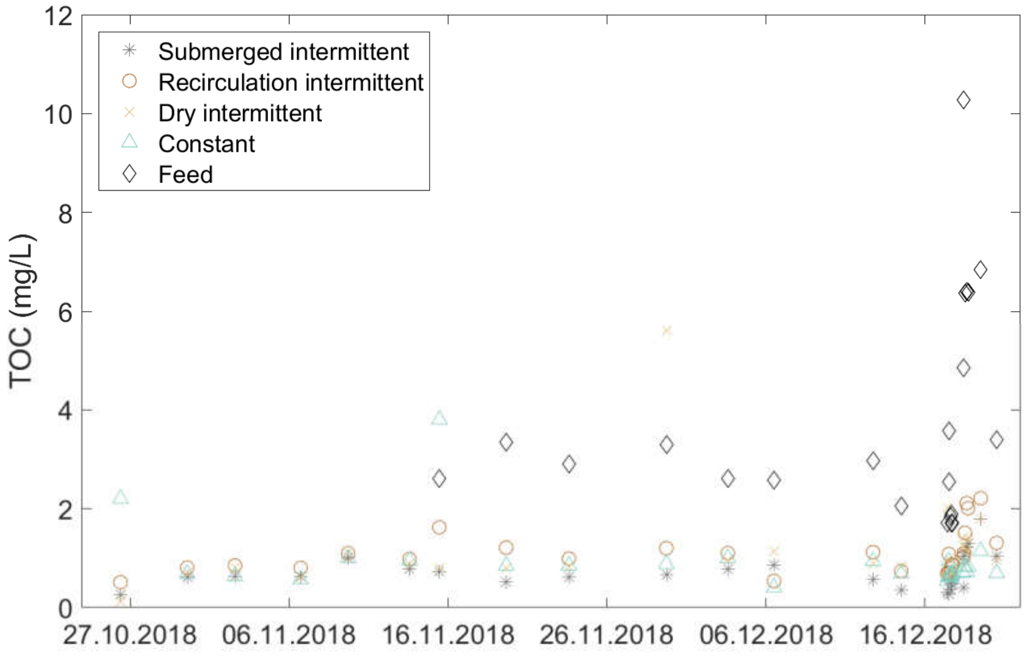

Figure S2: TOC concentrations over time from the feed and the effluent of the four small-scale columns.

2.2 Experiment 2

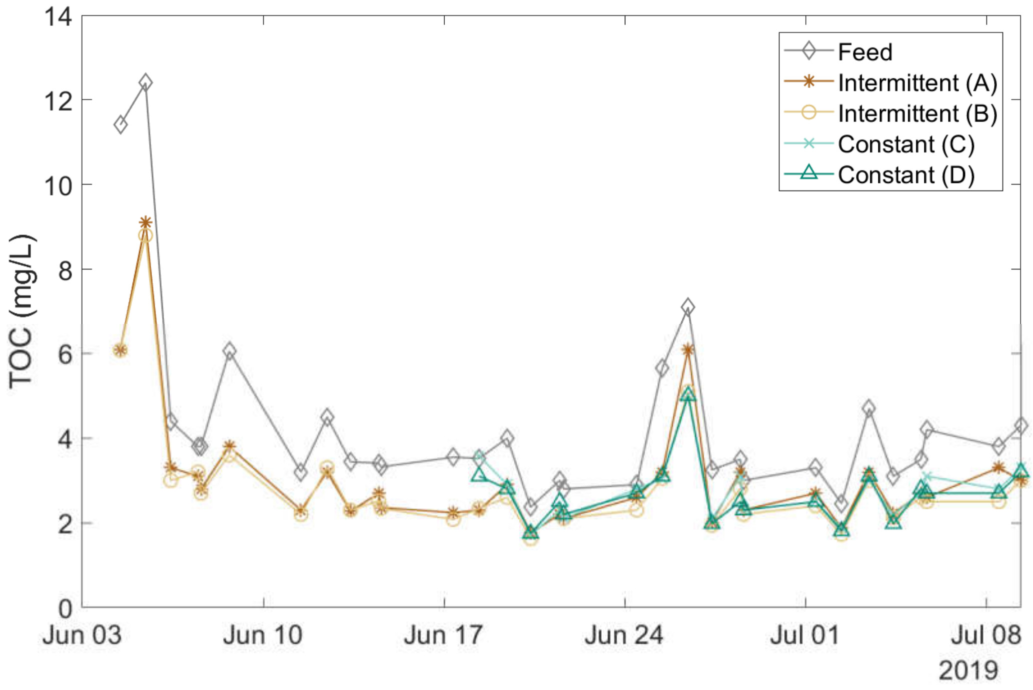

Figure S3: TOC concentrations over time during normal influent concentrations for the feed and the effluent of the four columns.

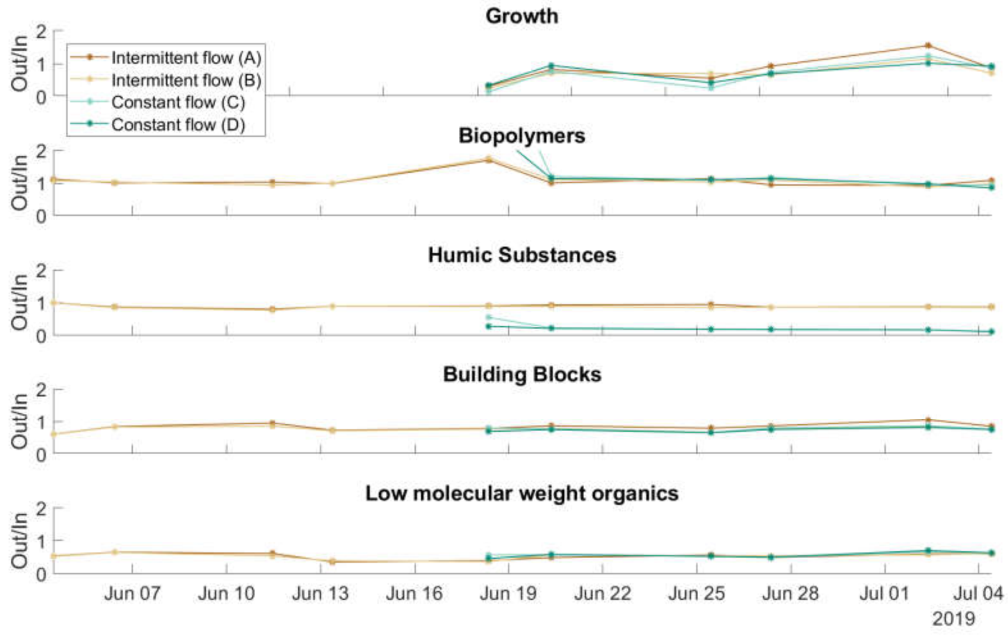

Figure S4: Growth potential and the different TOC fractions over time for the time with normal influent concentrations.

S 3 SEC chromatograms

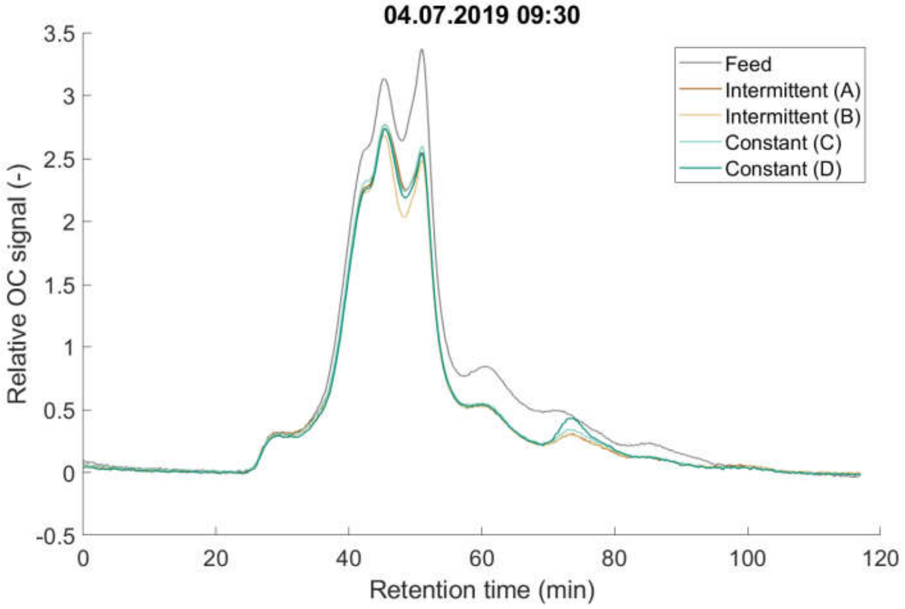

Figure S5: Typical chromatogram during times of normal influent concentrations.

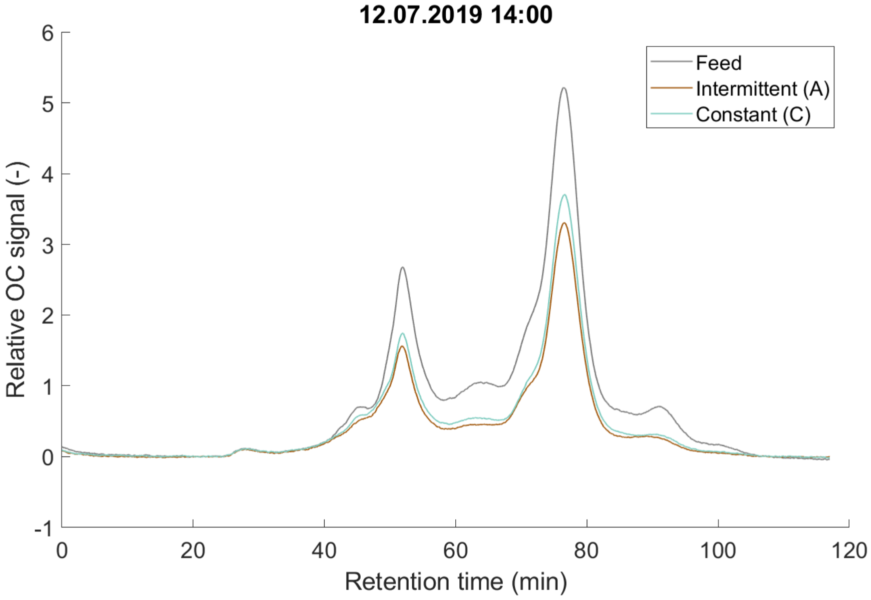

Figure S6: Sample chromatogram during high influent concentrations.

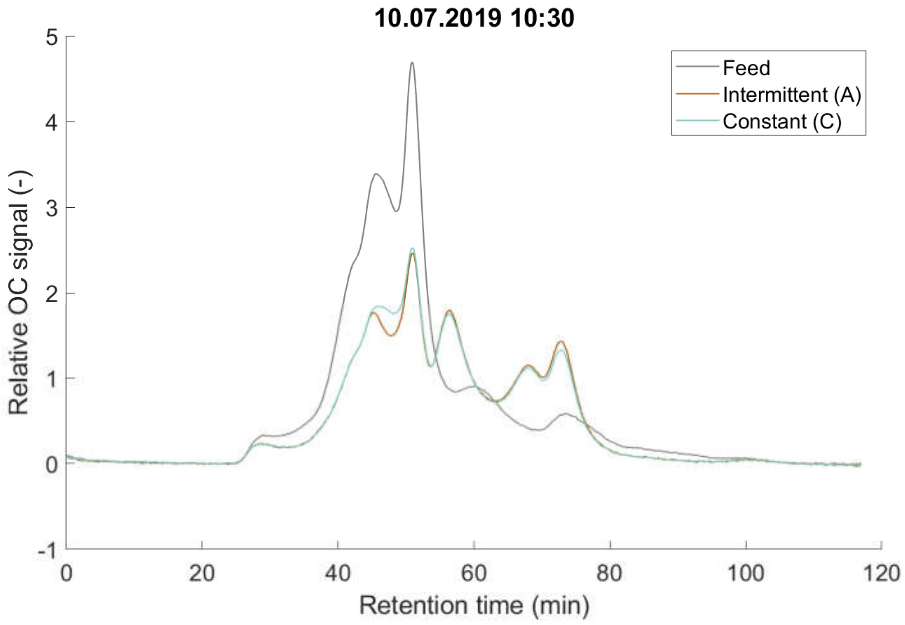

Figure S7: Sample chromatogram after switching back the high influent to normal influent concentrations.

S 4 ATP calibration

For the calibration, sterilized GAC (autoclaved) was treated like GAC usually is for ATP analysis (sonicated 3 times) and then know amount of ATP standard were added for calibration (Figure S8).

Each time when new ATP measurements were performed, one standard was measured to correct the calibration curve with this offset.

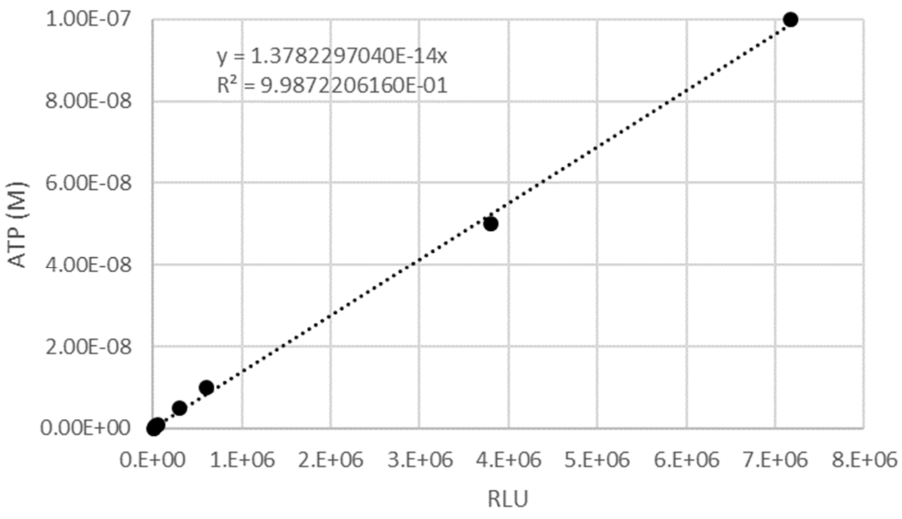

Figure S8: Calibration curve for ATP measurements on GAC.

S 5 ATP measurements

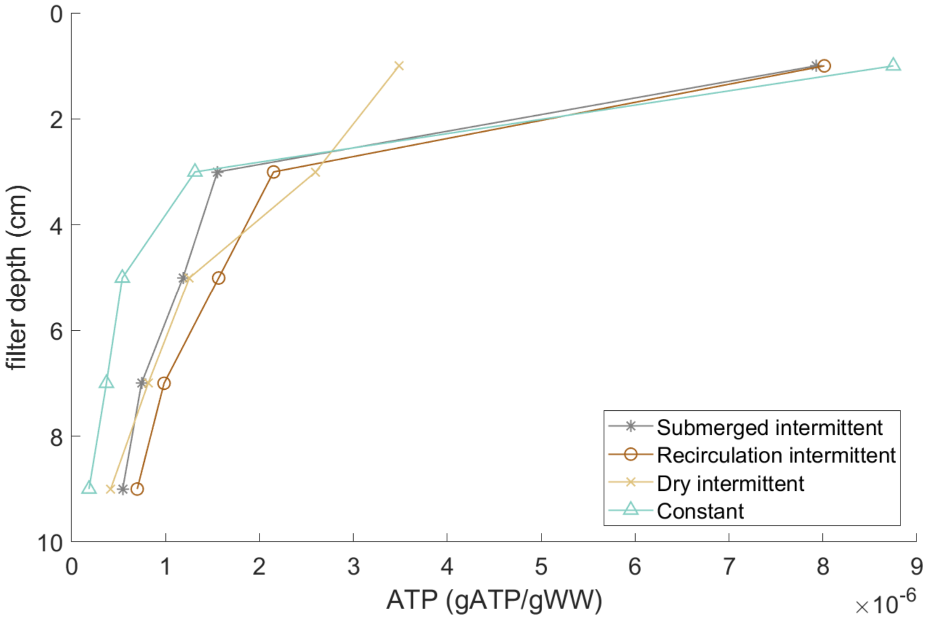

Figure S9: ATP measured over the filter bed for the four small-scale columns in experiment 1. A filter depth of 0 cm indicates the top of the filter bed.

S 6 Sorption capacity

The sensitivity of batch tests with methylene blue to characterize the sorption capacity of GAC was tested with GAC from the full-scale greywater treatment system. Virgin GAC was compared to exhausted GAC from three different depths of the filter bed (G1: 7 cm below surface, G2: 22 cm below surface, G3: 37 cm below surface). As control, a methylene blue solution without added GAC was used.

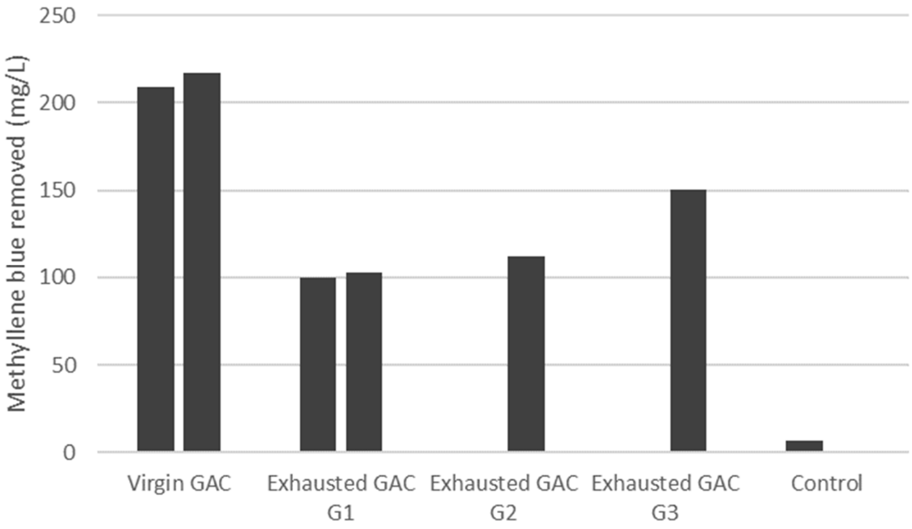

Figure S10: Methylene blue removed in batch tests to characterize the sorption capacity in the full-scale BAC for greywater treatment.
